# Supplementary material for: Augmentation of the Benzyl Isothiocyanate-Induced Antiproliferation by NBDHEX in the HCT-116 Human Colorectal Cancer Cell Line
Source: Int J Mol Sci. 2025 Aug 22;26(17):8145. doi: 10.3390/ijms26178145 (PMC12428750; doi:10.3390/ijms26178145)
Supplement: Supplementary file 1 [file ijms-26-08145-s001.zip › ijms-3697215-supplementary.pdf]

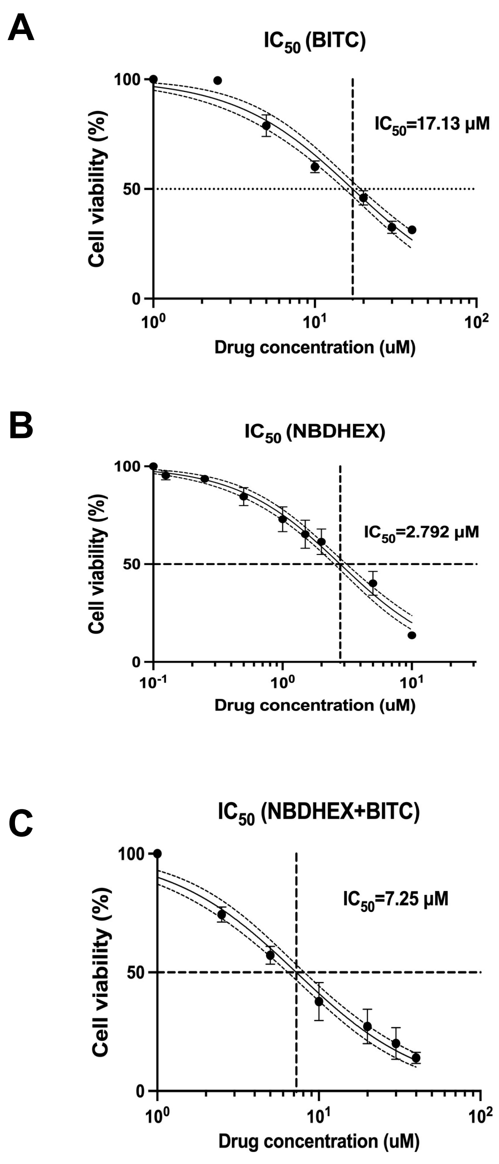

Figure S1. Concentration-dependent changes in viability of the HCT-116 cells treated with (A) BITC, (B) NBDHEX and (C) BITC and NBDHEX (combination with a molar ratio of 20:1). The solid line is the regression curve for the logistic function, and the dash line indicates the 95% confidence interval.
